# Di-Cyclohexene Oxide Bridged by DDSQ: Preparation, Characterization, and Application as Fillers for Cyanate Ester Resin

**DOI:** 10.3390/molecules30051113

**Published:** 2025-02-28

**Authors:** Xiaoye Gao, Wencheng Gao, Dingyi Lu, Riwei Xu

**Affiliations:** College of Materials Science and Engineering, Beijing University of Chemical Technology, Beijing 100029, China; m13785142844@163.com (X.G.); gaowencheng418@163.com (W.G.); 320586lu@gmail.com (D.L.)

**Keywords:** POSS, nanocomposites, dielectric properties, cyanate ester resin

## Abstract

In order to improve the dielectric properties of existing thermosetting resins, taking advantage of reactive fillers is a simple and feasible option. In this paper, we synthesized a new double epoxycyclohexane double-decker silsesquioxane (DEDDSQ), in which the structure of aliphatic epoxy resin introduced into DDSQ successfully, and the resulting structure of DEDDESQ is confirmed by Fourier transform infrared (FTIR), nuclear magnetic resonance (NMR) spectroscopy, and mass spectrometry (MS). Cyanate ester resin was selected as the case study for the application of DEDDSQ as reactive fillers. A CE/E51/DEDDSQ nanocomposite was fabricated by incorporating a small proportion of E51 resin and DEDDSQ into cyanate ester resin to enhance its comprehensive properties. X-ray diffraction (XRD) and energy-dispersive spectroscopy (EDS) analyses demonstrated that DEDDSQ dispersed uniformly within the resin matrix. Dynamic mechanical analysis (DMA) demonstrated that the CE/E51/8.0DEDDSQ nanocomposites exhibit excellent thermal properties. The glass transition temperature (*T_g_*) of the nanocomposite was measured to be 264 °C, indicating its excellent thermal stability. Dielectric property measurements showed that the addition of DEDDSQ reduced the dielectric constant of the cyanate ester resin, with the CE/E51/8.0DEPOSS nanocomposite exhibiting a dielectric constant of 2.47 at 1 MHz.

## 1. Introduction

With the rapid development of 5G technology, the Internet of Things, and smart electronic devices, the demand for high-performance materials is growing, and how to transmit fast and high capacity at high frequencies has become a challenge to be overcome [1]. Thermoset resins are widely used in many fields, such as electronics, electrical, and aerospace, due to their excellent mechanical properties, thermal stability, and processing flexibility; the common ones are epoxy resins [2], phthalonitrile resins [3], and cyanate ester resins [4]. However, with the advancement of communication technology, the dielectric properties of these conventional resins in high-frequency environments have become inadequate to meet the increasingly demanding requirements. Cyanate ester (CE) resin, as a kind of thermosetting resin, has advantages such as a low dielectric constant and excellent thermal stability, which has important application potential in high-frequency electronic devices, but is still insufficient to satisfy the demands of present-day communication equipment [5,6,7,8,9]. The study of how to further reduce the dielectric constant and dielectric loss of cyanate ester resins through material modification, while maintaining their excellent overall performance, has become one of the active research topics in the field of materials science [10,11].

Polyhedral oligomeric silsesquioxanes (POSSs) are hybrid nanostructures with the general formula (RSiO_1.5_)*_n_* or T_*n*_, where *n* represents the number of repeating units (*n* = 6, 8, 10 or 12, T = RSiO_1.5_), and R can be hydrogen or organic functional groups, either reactive or inert [12,13,14,15,16]. POSS molecules have a well-defined cubic core with R groups attached to their vertices, forming hybrid inorganic–organic cage-like structures. With diameters of 1–3 nm, POSS is considered the smallest silica particle [17,18,19]. POSS has been widely applied to polymers to enhance their overall performance for several reasons: (1) POSS molecules often carry reactive functional groups that can chemically bond with polymers. As POSS is inherently a nanoparticle, it disperses well in polymer matrices, addressing the issue of uneven filler dispersion [20,21,22]. (2) POSS molecules, as hollow cage-like structures (dielectric constant of air = 1), provide a large free volume, significantly reducing the dielectric constant of the host polymer [23,24,25,26]. (3) The Si-O-Si inorganic framework of POSS enhances the thermal stability of the composite material [27,28,29]. Given these attributes, incorporating POSS into low-dielectric materials is of great interest [30,31,32].

In recent years, based on the properties possessed by POSS, more and more scientists have applied POSS in the preparation of low-dielectric and low-loss materials [33,34]. Liu et al. [35] used aminopropylisobutyl-POSS to react with hydroxyl-containing polyimide (PI) to obtain a series of PI–POSS hybrids, which, compared with unmodified PI, showed dielectric constant and loss reductions of ~27% (2.68 vs. 3.68) and ~72% (0.007 vs. 0.025), respectively, compared to unmodified PI. Zhang et al. [36] prepared novel eugenol-functionalized POSS (EG-POSS) copolymerized with bismaleimide resin, and the dielectric constant (k) and loss (tan δ) at 1 MHz were reduced from 3.33 and 0.013 to 2.88 and 0.010. Periyasamy et al. [37] obtained an ultra-low dielectric constant nanocomposite by mixing eugenol-based benzoxazine monomers with octaamino POSS (OAPs), and the nanocomposite with 5 wt% of OPAs had a dielectric constant of 1.32 at 1 MHz frequency. For the application of epoxy-based POSS, Li et al. [38] obtained E51/BADCy/EOVS nanomaterials by blending octa-epoxy POSS (EOVS) after octa-vinyl POSS (OVS) epoxidation with epoxy resin/cyanate ester mixtures (mass fraction 1:1), and the dielectric constant and dielectric loss even decreased to 2.0 and 0.0036 (1 MHz). Wang et al. [39] added 40% CE to epoxy resin along with 0.4% glycidyl POSS (EP-POSS) to produce nanocomposites with *T_g_* up to 263 °C. To the best of our knowledge, most of the synthesis and application of epoxy-based POSS are dominated by T8 or a mixture of POSS, and few double-decker silsesquioxane-type (DDSQ-type) epoxy compounds with accurate molecular structures have been reported. Zhang et al. [40] synthesized a 3,13-diglycidoxypropyl octaphenyl double-decker silsesquioxane, which was applied to polybenzoxazinium (PBZ) thermosetting resins, and the thermal and mechanical properties were significantly improved. Sun et al. [41] synthesized a new type of bifunctional epoxy compound (DDSQ-EP) based on double-decker silsesquioxane (DDSQ), and doped it into polybenzoxazine (PBZ), and the results showed that residual carbon filtration was improved by 14% after the addition of DDSQ-EP.

In this study, a novel polyhedral oligomeric silsesquioxane (POSS) was synthesized to enhance the properties of thermosetting resins, with cyanate ester resin selected as a representative system for investigation. 4-vinyl epoxycyclohexane was grafted onto DDSQ via a hydrosilylation addition reaction, yielding a novel double epoxycyclohexane double-decker silsesquioxane (DEDDSQ), which was subsequently incorporated into alicyclic epoxy resins and subjected to comprehensive structural characterization. Subsequently, CE/E51 resin (mass ratio 8:2) was utilized as the matrix, modified by the addition of DEDDSQ to produce cyanate ester nanocomposites. The resulting nanocomposites were extensively studied to evaluate their low dielectric, thermal, and mechanical properties as influenced by the incorporation of DEDDSQ and organic polymers.

## 2. Results and Discussion

### 2.1. Characterizations of DEDDSQ

**Figure 1:**
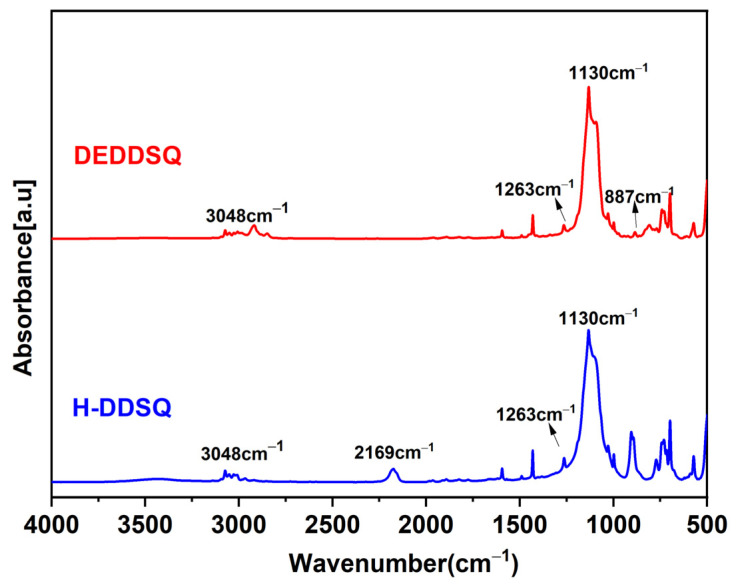
FT-IR spectrum of DEDDSQ.

Figure 1 shows the infrared spectra of H-DDSQ and DEDDSQ. By comparing the two spectra, the characteristic peak of Si-H disappeared at 2169 cm^−1^, while the characteristic peak of the epoxy group appeared at 887 cm^−1^. Additionally, the stretching vibration peak of Si-CH_3_ appeared at 1263 cm^−1^, the stretching vibration peak of Si-O appeared at 1130 cm^−1^, and the characteristic peak of the benzene ring appeared at 3048 cm^−1^. These observations indicate that DEDDSQ was successfully synthesized.

**Figure 2:**
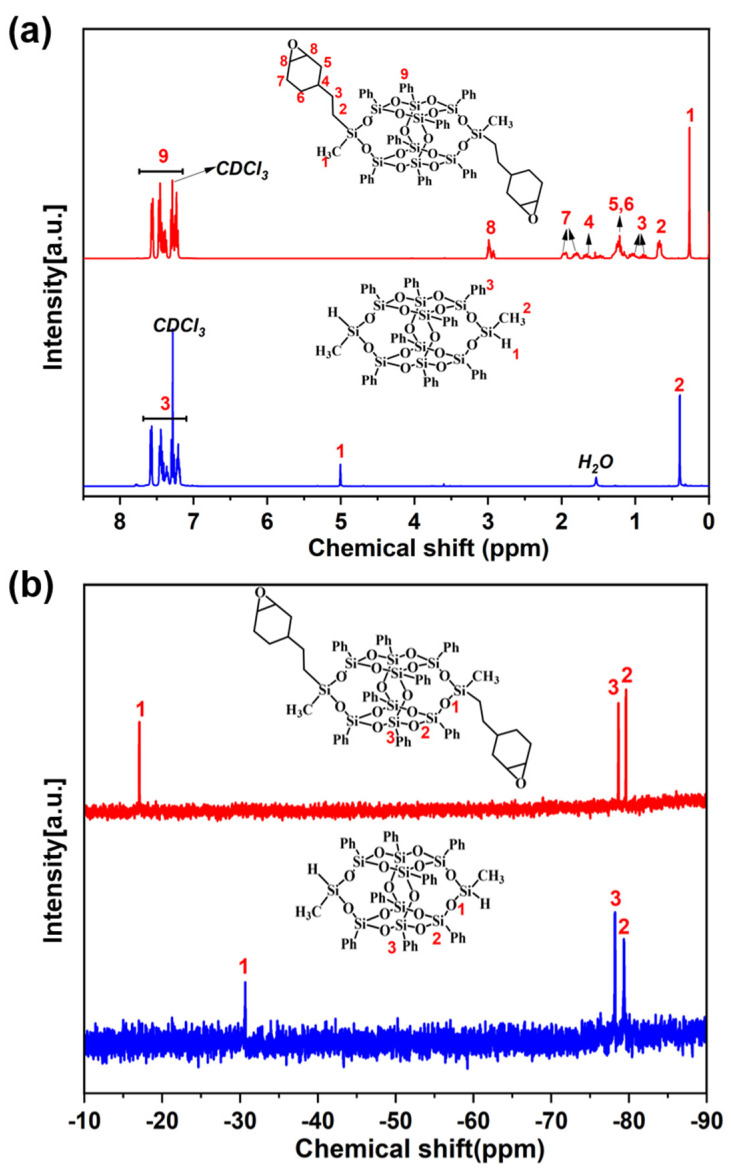
NMR characterization of DEDDSQ: (**a**) ^1^H-NMR spectrum and (**b**) ^29^Si NMR spectrum. (Red represents DEDDSQ, blue represents H-DDSQ, and the numbers indicate the assignments of their proton peaks respectively).

Figure 2a presents the ^1^H NMR spectra comparing H-DDSQ and DEDDSQ. In the spectrum of DEDDSQ, H1 (0.31 ppm) corresponds to the proton peak of Si-CH_3_, H8 (2.97–3.02 ppm) corresponds to the proton peak of the hydrogen on the epoxy group, H2 (0.72 ppm) and H3 (1.08 ppm) correspond to the proton peaks of two methylene groups close to silicon, H4–H8 (1.26–3.02 ppm) correspond to the proton peaks of hydrogens on the cyclohexane ring, and H9 (7.20–7.59 ppm) corresponds to the proton peaks of hydrogens on the benzene ring. The ratio of peak areas, SH1:SH2:SH3:SH4:SH5 + SH6:SH7:SH8 = 3:2.19:1.88:1.21:4.04:2.13:2.04, closely matches the theoretical integral ratio of 3:2:2:1:4:2:2, confirming the successful synthesis of the product. Compared to the spectrum of H-DDSQ, the signal peak of Si-H at 5.01 ppm disappeared, further indicating that the reaction was complete.

The ^29^Si NMR spectrum of DEDDSQ is shown in Figure 2b. In this spectrum, δ = −17.05 ppm corresponds to the characteristic peak of Si1, δ = −78.65 ppm corresponds to the characteristic peak of Si3, and δ = −79.61 ppm corresponds to Si2. Compared with the silicon spectrum of H-DDSQ, the chemical shifts of silicon have changed, which is attributed to the structural differences in the substituents.

**Figure 3:**
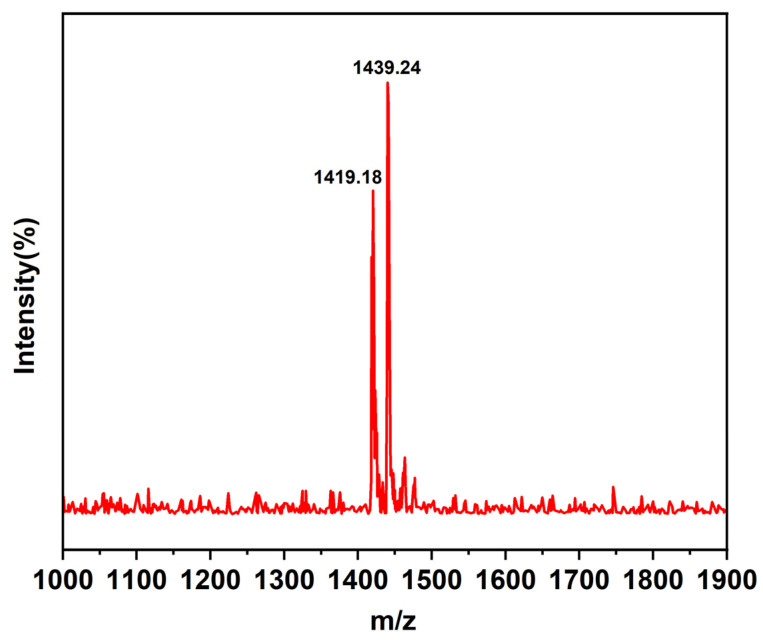
Mass spectrum of DEDDSQ.

The liquid chromatography–mass spectrometry (LC–MS) results for DEDDSQ are shown in Figure 3. The peaks at 1419.18 (M + H_2_O^+^, 1400 + 19) and 1439.24 (M + K^+^, 1400 + 39) are consistent with the expected molecular weight. This agreement between the measured and theoretical molecular weights confirms the successful synthesis of the product.

### 2.2. Curing Mechanism of CE/E51/DEDDSQ Resin

**Figure 4:**
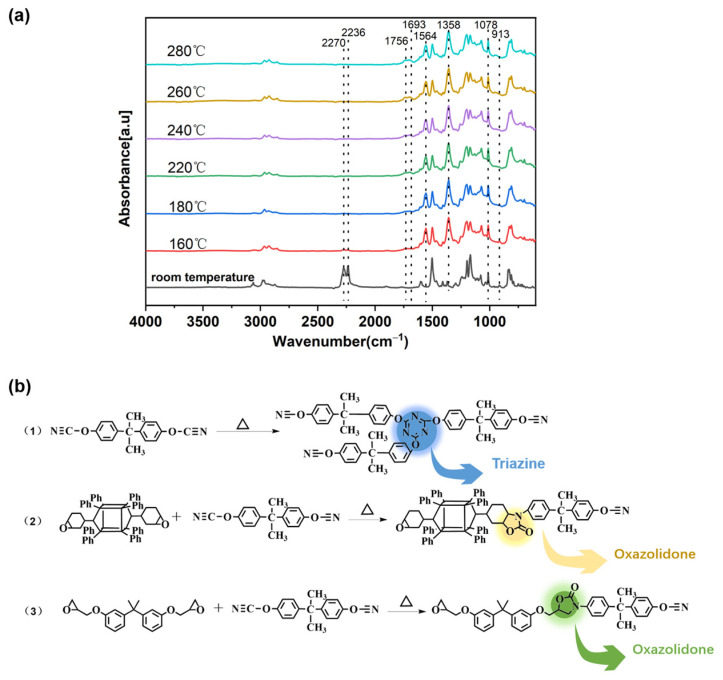
(**a**) FT-IR spectra of CE/E51/DEDDSQ at different temperatures in the curing process and (**b**) main reactions of CE/E51/DEDDSQ resin: (1) Self-aggregation of CE; (2) Reaction between DEDDSQ and CE; (3) Reaction between E51 and CE.

FT-IR was employed to characterize the cured products at each stage of stepwise process of curing, as illustrated in Figure 4a. The FT-IR spectra of CE/E51/DEDDSQ resins during each stage of the curing process are presented in the figure. The characteristic infrared absorption peaks of cyanate esters are located at 2236 cm^−1^ and 2270 cm^−1^. When the temperature was increased to 160 °C the absorption peak of the cyanate ester nearly disappeared, while the characteristic peaks of the triazine ring appeared at 1358 cm^−1^ and 1564 cm^−1^, indicating that the CE monomer underwent self-polymerization. Starting at 180 °C, as the temperature increased, the characteristic peak of oxazolidone at 1756 cm^−1^ emerged and intensified, while the characteristic peak of epoxy at 913 cm^−1^ diminished. Additionally, the characteristic peak of Si-O-Si at 1078 cm^−1^ confirmed that DEDDSQ fully participated in the reaction. In summary, the reaction for the curing process of CE/E51/DEDDSQ resin primarily involves two reactions. First, cyanate esters undergo self-polymerization to form triazine rings. This reaction is largely complete at 160 °C, demonstrating the high reactivity of cyanate esters. The second reaction involves the reaction between epoxy groups and cyanate esters, yielding oxazolidinone. This process is essentially complete at 260 °C.

The proposed reaction mechanism is illustrated in Figure 4b. The cross-linking structures in the resins predominantly consist of triazine rings and oxazolidinone groups. Notably, the cyclohexyl epoxy groups exhibit low reactivity; however, the high reactivity of cyanate esters ensures the formation of a well-defined cross-linking structure. DEDDSQ containing two epoxy groups not only effectively participates in the polymer cross-linking network but also avoids excessive epoxy functionality. With the large structure in DEDDSQ, the free volume increased, which is advantageous for lowering the material’s dielectric constant.

### 2.3. Curing Behavior and Curing Kinetics of CE/E51/DEDDSQ Resin

**Figure 5:**
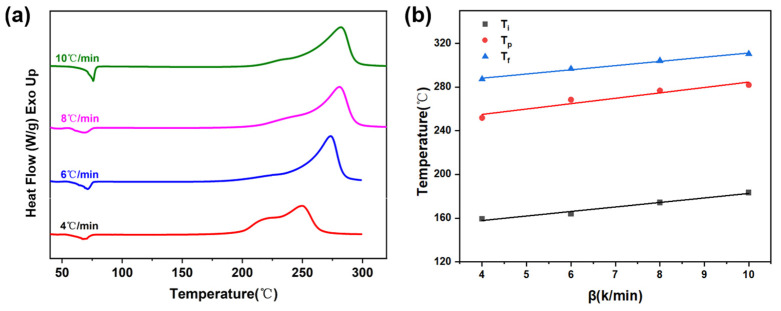
(**a**) DSC curves of CE/E51/8.0DEDDSQ at different heating rates; (**b**) Heating rate temperature curve of CE/E51/DEDDSQ obtained by extrapolation method.

The curing kinetics of CE/E51/8% DEDDSQ resin was investigated. Non-isothermal curing DSC curves were recorded at heating rates of 10 °C/min, 8 °C/min, 6 °C/min, and 4 °C/min, as shown in Figure 5a. Table 1 summarizes the non-isothermal curing DSC parameters of CE/E51/8% DEDDSQ resin at various heating rates. Here, β represents the heating rate, T_i_ is the initial curing temperature, T_p_ is the peak curing temperature, T_f_ is the end curing temperature, ΔT is the curing range, and ΔH is the curing exothermic enthalpy. As the heating rate increases, T_i_, T_p_, T_f_, and ΔH all exhibit an upward trend. Linear fits of T_i_, T_p_, and T_f_ against different heating rates (β) were obtained, as shown in Figure 5b. The slopes of the three curves were 4.13, 4.93, and 3.85, and the intercepts were 141.24, 235.24, and 272.74, and R^2^ were 0.9896, 0.9867, 0.9953, respectively. Intercept is the extrapolation value of T_i_, T_p,_ and T_f_ at β = 0. The curing temperature is determined according to the concept of fitting curve, in which T_i_, T_P,_ and T_f_ represent the theoretical initial curing temperature, peak curing temperature, and end curing temperature, respectively, which can be used as the reference value for the curing process. Based on these results and prior experimental experience, the curing procedure for CE/E51/DEDDSQ was optimized as follows: 160 °C for 1 h, 180 °C for 2 h, 220 °C for 2 h, 240 °C for 2 h, and 260 °C for 2 h.

The study of the curing kinetics of the CE/E51/8% DEDDSQ was continued using the data obtained from the characterization of the non-isothermal DSC curves. It is assumed that the cure reaction rate of the composites conforms to the Arrhenius Equation (1):

(1) $$\frac{d\alpha }{dt}=A\;exp\left(\frac{-E}{RT}\right){\left(1-\alpha \right)}^{n}$$

where *dα*/*dt* is the cure reaction rate, *A* is the pre-exponential factor, *E* is the apparent activation energy, *R* is the gas constant, *T* is the Kelvin temperature, and *n* is the number of reaction stages.

The Kissinger and Ozawa equations were used to find the respective activation energies, and then the average of the two was taken as the apparent activation energy, *E*. The activation energy was calculated using the Kissinger equation and the Ozawa equation, respectively. The Kissinger equation is shown in Equation (2):

(2) $$\frac{dln\left(\frac{\beta }{{T_{p}}^{2}}\right)}{d\left(\frac{1}{T_{p}}\right)}=-\frac{E}{R}$$

The Kissinger activation energy can be calculated by fitting *ln*(*β*/*T_p_*^2^) linearly to 1/*T_p_* and finding the slope of the resulting line. The Ozawa equation is shown in Equation (3):

(3) $$\frac{dln\beta }{d\left(\frac{1}{T_{p}}\right)}=-1.052\frac{E}{R}\;$$

The Ozawa activation energy can be calculated by fitting *lnβ* linearly to 1/*T_p_* and finding the slope of the resulting line.

**Figure 6:**
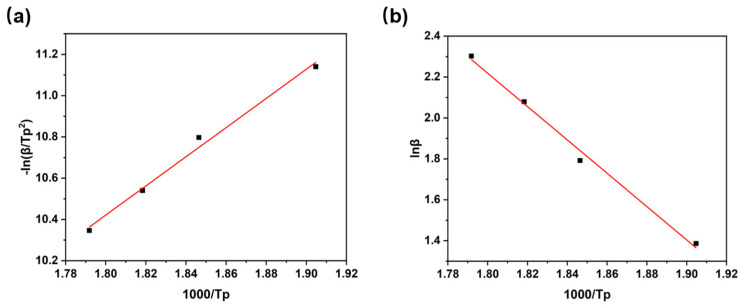
Linear fitting curves of CE/E51/8.0DEDDSQ resin: (**a**) Kissinger equation and (**b**) Ozawa equation.

The data required for the linear fit are listed in Table 2, Figure 6a shows the linear fit to the Kissinger equation, and Figure 6b shows the linear fit to the Ozawa equation. The slopes of the two curves are 7.07 and −8.15, and the intercepts are −2.30 and 16.89, and *R*^2^ are 0.9956 and 0.9932, respectively. The calculated Kissinger activation energies, Ozawa activation energies, and mean activation energies are listed in Table 3.

We used the Crane equation to determine the number of reaction stages *n*. The Crane equation is shown in Equation (4):

(4) $$\frac{dln\beta }{d\left(\frac{1}{T_{p}}\right)}=-\frac{E}{nR}+2T_{p}$$

The number of reaction stages *n* can be calculated by taking the mean activation energy obtained from the previous calculations as *E* and then taking the slope obtained from the linear fit of the Ozawa equation; the results are listed in Table 3.

We used Equation (5) to calculate the pre-exponential factor *A*:

(5) $$A=\frac{\beta Eexp\left(\frac{E}{RT_{p}}\right)}{RT_{p}^{\;2}}\;$$

The relevant calculation results are shown in Table 4.

We used the curing kinetic equation for CE/E51/8%DEDDSQ with a reaction level closer to the primary reaction. The solidification kinetics equation is shown in Equation (6).

(6) $$\frac{d\alpha }{dt}=2.06\times 10^{5}\exp\left(\frac{-63,260}{RT}\right){\left(1-\alpha \right)}^{1.07}$$

### 2.4. Surface Topography Analysis

**Figure 7:**
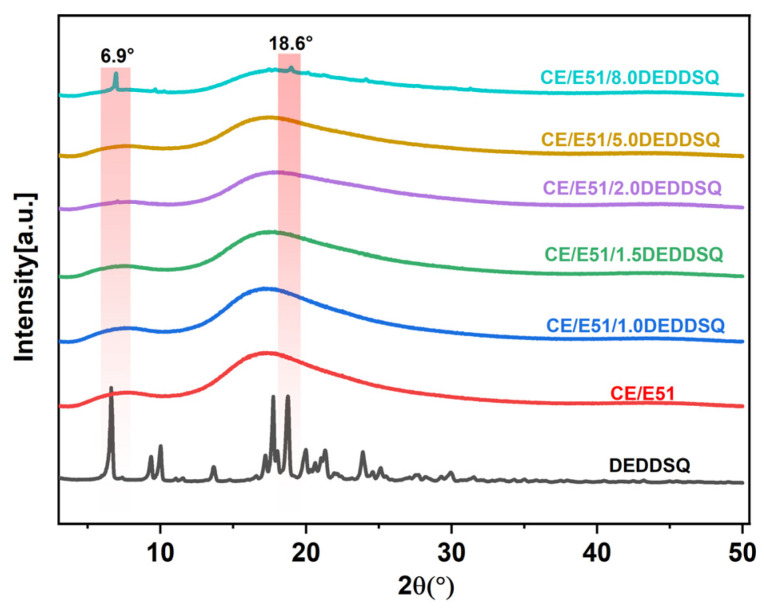
XRD spectra of CE/E51/DEDDSQ nanocomposites.

The uniform dispersion of the filler within the resin matrix is crucial for the performance of the nanocomposites. X-ray diffraction (XRD) was used to evaluate the compatibility between DEDDSQ and the resin matrix. As shown in Figure 7, the XRD patterns of nanocomposites containing 1%, 1.5%, 2%, 5%, and 8% DEDDSQ exhibited a single dispersion peak at 2θ = 18.5°, indicating excellent compatibility between DEDDSQ and the resin matrix. However, when the DEDDSQ content reached 8%, the XRD spectra showed weak diffraction peaks at 2θ = 6.9° and 18.6°, respectively, suggesting that the dispersion compatibility of DEDDSQ in the resin began to decline. This observation indicates that 8% DEDDSQ represents the upper limit of dispersion in this system, beyond which dispersion deteriorates. Therefore, 8% DEDDSQ was identified as the maximum compatible addition for this system.

**Figure 8:**
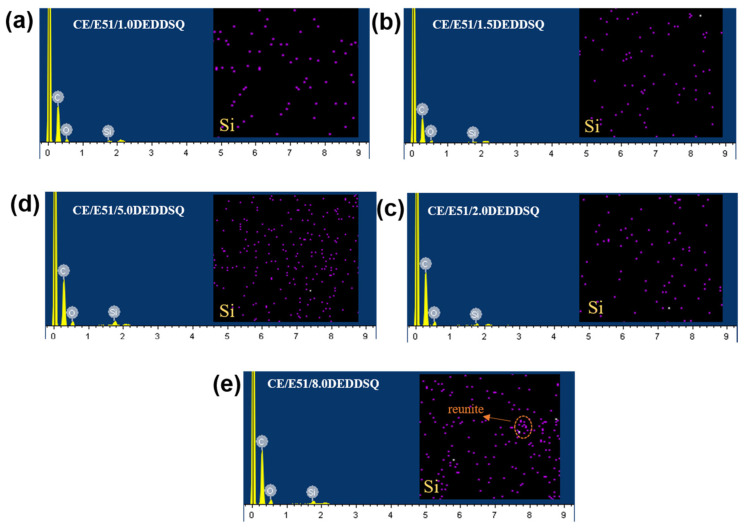
EDS of CE/E51/DEDDSQ nanocomposites.

Energy-dispersive spectroscopy (EDS) provided intuitive insights into the silicon content and the dispersion of POSS nanoparticles within the matrix. As shown in Figure 8, the EDS spectrum revealed a gradual increase in silicon content as the DEDDSQ content increased. The Si-mapping images demonstrated the uniform dispersion of DEDDSQ nanoparticles without aggregation at POSS contents of 1%, 1.5%, 2%, and 5%. At 8% DEDDSQ content (Figure 8e), slight nanoparticle agglomeration was observed. This phenomenon can be attributed to DEDDSQ forming covalent bonds with cyanate ester groups, facilitated by the dispersion effect of E51. This interaction enhanced the compatibility of DEDDSQ nanoparticles within the resin matrix, confirming that uniform dispersion was achievable up to 8% DEDDSQ content.

### 2.5. Thermal Performance Analysis

**Figure 9:**
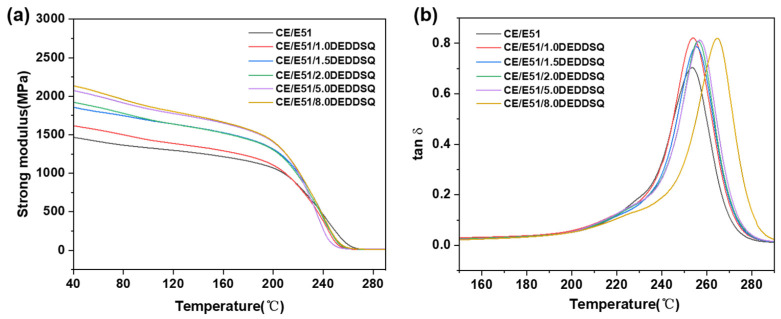
(**a**) Storage modulus and (**b**) tan δ for CE/E51/DEDDSQ nanocomposites.

Heat resistance is a critical property for materials used in electronic and electrical applications. The storage modulus (E’) and glass transition temperature (*T_g_*) of the nanocomposites were evaluated using dynamic mechanical analysis (DMA). The results are illustrated in Figure 9a. The E’ represents the material’s stiffness. A higher storage modulus indicates greater stiffness and enhanced resistance to deformation. As the temperature increased, E’ decreased gradually; however, when the temperature reached approximately 200 °C, a sharp decline in E’ was observed. When the POSS content was 0%, the initial storage modulus was 1467 MPa. As the POSS content increased, E’ also increased, primarily due to the macroscopic effects of DEDDSQ nanoparticles. DEDDSQ fully integrated into the resin’s crosslinking network. When the DEDDSQ content reached 8%, E’ increased to 2135 MPa, indicating the formation of more rigid network structures, which enhanced the rigidity and deformation resistance of the nanocomposites. Regarding *T_g_*, as shown in Figure 9b, it was measured at 253 °C for nanocomposites with 0% DEDDSQ. As the DEDDSQ content increased, *T_g_* also increased. For DEDDSQ contents of 1%, 1.5%, and 5% the *T_g_* increase was marginal, with an increase of less than 10 °C compared to 0% DEDDSQ. However, at 8% DEDDSQ content, *T_g_* increased significantly, reaching 264 °C. This significant *T_g_* enhancement can be attributed to the increased DEDDSQ content, which restricts the mobility of molecular chains. The addition of DEDDSQ significantly enhances the dynamic mechanical properties of the composites.

**Figure 10:**
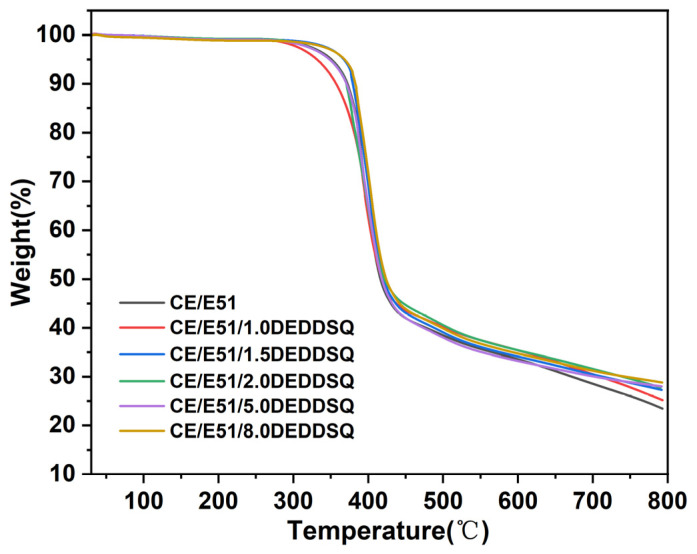
TGA curve of CE/E51/DEDDSQ nanocomposites.

Figure 10 presents the thermal weight loss (TGA) curves of CE/E51/DEDDSQ nanocomposites. The decomposition temperature at 10% weight loss and the char yield at 800 °C are summarized in the table. The data in the table and the trend of the curve indicate that the decomposition temperature at 10% weight loss for CE/E51 is 368 °C. After incorporating various proportions of DEDDSQ, the decomposition temperature of CE/E51/DEDDSQ nanocomposites at 10% weight loss showed a gradual increase. The char yield for CE/E51/0% DEDDSQ at 800 °C was 24.3%. The char yield of the nanocomposites increased proportionally with the addition of DEDDSQ. As shown in Table 5, the nanocomposites with an 8% DEDDSQ content exhibited the highest char yield of 29.1%, while the maximum thermogravimetric decomposition temperature reached 402.6 °C. This enhancement is attributed to the higher DEDDSQ content. This improvement can be attributed to the increased DEDDSQ content. The TGA results demonstrate that the incorporation of DEDDSQ enhanced the resin’s thermal stability and high-temperature resistance. This enhancement can be attributed to the well-dispersed DEDDSQ and the thermal stability conferred by the Si-O-Si structure.

### 2.6. Dielectric Properties Analysis

**Figure 11:**
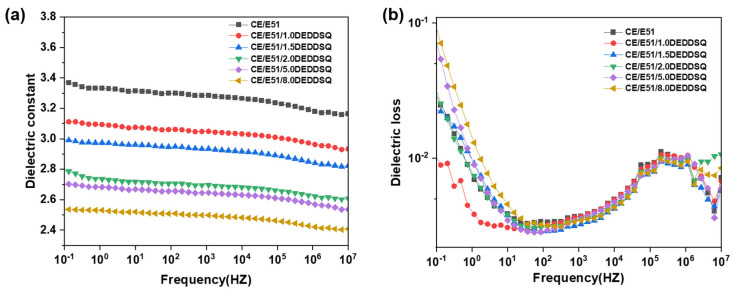
(**a**) Dielectric constant of CE/E51/DEDDSQ nanocomposites and (**b**) dielectric loss of CE/E51/DEDDSQ nanocomposites.

Cyanate ester resin is widely used in microelectronics and other high-performance applications, where its dielectric constant and dielectric loss are critical properties. Figure 11a,b present the dielectric constant and dielectric loss of CE/E51/DEDDSQ nanocomposites, respectively. Regarding the dielectric constant, it is evident that with increasing DEDDSQ content, the dielectric constant decreases over the frequency range of 10^−1^ to 10^7^ Hz. Moreover, every sample exhibits minimal fluctuation in the dielectric constant across this frequency range. Specifically, the dielectric constant of CE/E51 resin is 3.24 at 1 MHz, whereas that of CE/E51/8.0DEDDSQ nanocomposites decreases to 2.47 at 1 MHz when the DEDDSQ content reaches 8 wt%. In terms of dielectric loss, all samples exhibit a trend of initial decrease, followed by an increase, and then a subsequent decrease. The reduction in dielectric loss below 10^2^ Hz is primarily attributed to the influence of impurities and interfacial polarization, while the variations beyond 10^2^ Hz are associated with the presence of polar groups, such as oxazolidinone and triazine rings, in the system [42]. Overall, compared with CE/E51 resin, the incorporation of DEDDSQ has a negligible impact on the dielectric loss, with no significant changes observed. The dielectric loss of CE/E51 resin is 0.0096 at 1 MHz, while that of CE/E51/8.0DEDDSQ nanocomposites is 0.0088 at 1 MHz when the DEDDSQ content reaches 8 wt%. The incorporation of DEDDSQ effectively reduces the dielectric constant of the nanocomposites, while the dielectric loss remains largely unchanged. This can be explained by the following factors. First, cyanate ester resin forms triazine ring structures during curing, showing low polarity, which helps to reduce the dielectric constant. Second, the introduction of DEDDSQ with a hollow cage structure increases the free volume in the resin, which reduces the density of the material and further reduces the dielectric constant. Third, the cyclohexane group grafted on DDSQ has a large steric hindrance, which may further increase the nanocomposites. Therefore, considering the aforementioned three factors, the resulting CE/E51/DEDDSQ-cured product exhibits excellent dielectric properties.

### 2.7. Moisture Resistance Analysis

**Figure 12:**
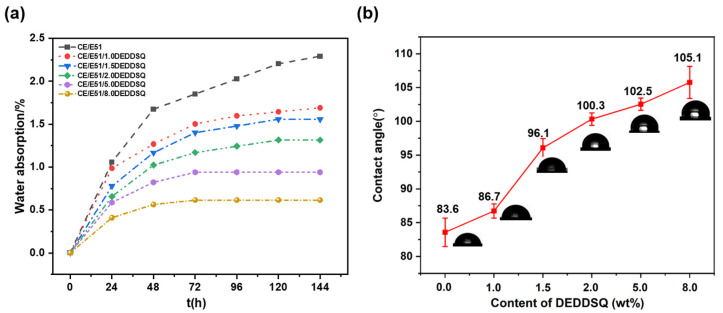
(**a**) Water absorption of CE/E51/DEDDSQ nanocomposites and (**b**) contact angles of CE/E51/DEDDSQ nanocomposites.

Moisture resistance of thermosetting resin is a critical factor influencing its dielectric properties. Figure 12a presents the results of a 144 h continuous test on resin water absorption. During the test, water molecules gradually diffused from the surface to the interior of the resin. The water absorption rates of resins with different proportions of DEDDSQ were markedly different. Without the addition of DEDDSQ, the water absorption rate reached 2.29% after 144 h. However, the addition of DEDDSQ significantly reduced water absorption, with the CE/E51/8.0DEDDSQ nanocomposites exhibiting a water absorption rate of only 0.49%. All resins showed a uniform increase in water absorption during the initial 1–72 h, followed by a plateau after 72 h. The water contact angle test results of CE/E51/DEDDSQ nanocomposites are illustrated in Figure 12b. The findings demonstrate that the amount of DEDDSQ significantly impacts the hydrophilicity and hydrophobicity of the nanocomposites. For the resin without DEDDSQ, the water contact angle was 83.6°, indicating a hydrophilic material. With the addition of DEDDSQ, the water contact angle increased. Notably, when the DEDDSQ content was 8 wt%, the water contact angle reached 105.1°. This improvement can be attributed to the low surface energy of the Si-O-Si inorganic framework in DEDDSQ, which enhances the water resistance of the nanocomposites.

## 3. Materials and Methods

### 3.1. Materials

Bisphenol A cyanate ester resin (CE), bisphenol A epoxy resin (E51), 1,3-divinyl-1,1,3,3-tetramethyldisiloxane platinum (0) (karstedt’s catalyst), acetonitrile, 1,2-epoxy-4-vinylcyclohexane, isopropanol, ultra-dry tetrahydrofuran, triethylamine, phenyltrimethoxysilane, sodium hydroxide, methanol, toluene, and dichloromethane were sourced from Beijing Huawei Ruike Chemical Technology Co. (Beijing, China). All reagents were used as received without further purification.

### 3.2. Characterization

Nuclear magnetic resonance (NMR) spectra were recorded using a Bruker ADVANCE 400 MHz spectrometer (Bruker, Billerica, MA, USA) with deuterated chloroform (CDCl_3_) as the solvent at 25 °C.

Fourier-transform infrared (FTIR) spectra were obtained using a Nicolet-670 spectrometer (Thermo Fisher Scientific, Waltham, MA, USA) over a wavenumber range of 4000–400 cm^−1^ with the potassium bromide (KBr) pellet method.

Mass spectrometry (MS) was performed on a UPLC/Premier system (Waters, Milford, MA, USA) in positive ion mode, with samples dissolved in tetrahydrofuran (THF).

Differential scanning calorimetry (DSC) measurements were carried out on a TA Instruments Q20 calorimeter (TA Instruments, New Castle, DE, USA) under a nitrogen flow rate of 50 mL/min. Approximately 5 mg of each sample was sealed in aluminum pans and tested at heating rates of 10, 8, 6, and 4 °C/min over a temperature range of 25–350 °C.

Thermogravimetric analysis (TGA) was conducted on a Rigaku TG-DTA 8122 system (Rigaku, Tokyo, Japan) under a nitrogen purge rate of 100 mL/min. Approximately 10 mg of sample was placed in a ceramic crucible and heated from 25 to 800 °C at a rate of 10 °C/min.

X-ray diffraction (XRD) was performed using a D/MAX 2500 VBZ+/PC instrument (Rigaku, Tokyo, Japan) with Cu-Kα radiation (λ = 0.154 nm). Scans were conducted at a speed of 5°/min over a range of 3–50°.

Energy-dispersive spectroscopy (EDS) was carried out on a Hitachi S4800 instrument (Hitachi, Chiyoda City, Japan).

Dynamic mechanical analysis (DMA) was conducted on a DMA Q800 instrument (TA Instruments, New Castle, DE, USA) in single cantilever mode at a frequency of 1 Hz, over a temperature range of 25–450 °C, with a heating rate of 5 °C/min. The sample dimensions were 40 mm × 10 mm × 2 mm.

Dielectric properties were measured using an Novocontrol Concept 80(Novocontrol GmbH, Montabaur, Germany). Sample dimensions were 20 mm in diameter and 1.5 mm in thickness. Measurements were conducted at 25 °C with an applied voltage of 3 V over a frequency range of 10^−1^–10^7^ Hz.

Water contact angle measurements were performed using a DSA100 instrument (KRÜSS GmbH, Hamburg, Germany) at 25 °C. Water absorption tests were performed at room temperature for the nanocomposites in accordance with ISO 62-2008 [43].

### 3.3. Synthesis of DEDDSQ

**Scheme 1:**
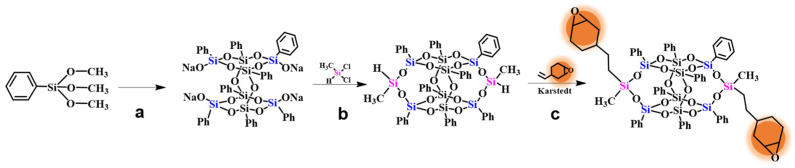
Synthesis route of DEDDSQ (a) synthesis of DDONa and (b) synthesis of H-DDSQ and (c) synthesis of DEDDSQ.

The synthetic route of DEDDSQ is shown in Scheme 1, where the synthesis of DDONa (Scheme 1a) and H-DDSQ (Scheme 1b) was carried out according to the literature [40]. The specific synthesis of DEDDSQ (Scheme 1c) was carried out as described below. Under nitrogen atmosphere, 6 g of H-DDSQ and 3.88 g of 1,2-epoxy-4-ethenylcyclohexane was taken and added to 50 mL of toluene, then added 10 μL of karstedt’s catalyst and stirred vigorously for 30 min to mix well. The temperature was raised to 90 °C, refluxed, and the reaction was carried out for 36 h. At the end of the reaction, most of the solvent was removed by spin distillation, dropped into acetonitrile, and a grey solid was precipitated, washed three times, and then put into an oven at 60 °C for 6 h. The product was obtained by drying, with a yield of 60%.

### 3.4. Synthesis of Nanocomposites

**Figure 13:**
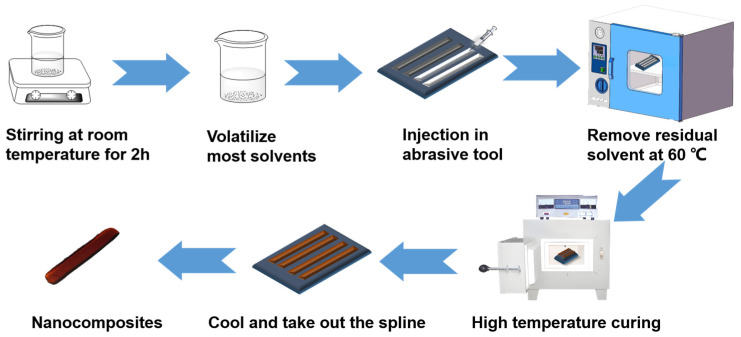
Schematic diagram of sample preparation of CE/E51/DEDDSQ nanocomposites.

The preparation process of CE/E51/DEDDSQ nanocomposites is shown in Figure 13, The DEDDSQ and CE/E51 (mass ratio: 8:2) were stirred and mixed in dichloromethane. The content of DEDDSQ was 0 wt%, 0.5 wt%, 1.0 wt%, 2.0 wt%, 5.0 wt%, and 8.0 wt%, respectively. After stirring for 2 h, most of the solvent was volatilized in the air, and the remaining solvent was dried in a vacuum oven to obtain the CE/E51/DEDDSQ nanocomposite mixture. The obtained mixture was cured and formed according to the curing process of 160 °C/2 h, 180 °C/2 h, 200 °C/2 h, 220 °C/2 h, 240 °C/2 h, and 260 °C/2 h, respectively, to obtain the CE/E51/DEDDSQ nanocomposite spline.

## 4. Conclusions

In this study, a novel double epoxycyclohexane double-decker silsesquioxane (DEDDSQ) was successfully synthesized and utilized to modify cyanate ester (CE) resin. The combined effects of the hollow cage structure of POSS and the alicyclic epoxy moieties significantly enhanced the comprehensive properties of the CE resin. Compared with CE/E51, the dielectric constant of CE/E51/8.0DEDDSQ nanocomposites was successfully reduced from 3.24 to 2.47 at 1 MHz. Regarding thermal performance, the glass transition temperature (*T_g_*) of CE/E51/8.0DEDDSQ nanocomposites increased notably by 15 °C, reaching 264 °C. In terms of moisture resistance, the water absorption of CE/E51/8.0DEDDSQ nanocomposites decreased by 1.8% to 0.49% after 144 h, while the water contact angle increased by 25.7%, reaching 105.1°.

In summary, this work successfully designed and developed a novel double-decker silsesquioxane containing double-epoxycyclohexane (DEDDSQ), which functioned as a reactive nanofiller to fabricate a CE-based nanocomposite with a well-balanced combination of thermal stability and dielectric properties. These findings provide valuable insights into the development of low-dielectric nanocomposites. It is anticipated that this reactive POSS could be further applied to other thermosetting resin systems, and further applied in microwave electronic packaging, aerospace composites, and low-dielectric substrates.

## Figures and Tables

**Table 1:** DSC parameters of CE/E51/8.0DEDDSQ at different heating rates.

β/°C/min	T_i_/°C	T_p_/°C	T_f_/°C	ΔT/°C	−ΔH/J/g
4	159.2	251.9	287.3	128.1	258.7
6	163.9	268.4	296.8	132.9	274.6
8	174.3	276.8	304.3	123.0	286.4
10	183.3	284.9	310.4	127.1	295.8

**Table 2:** Data required for linear fitting.

Samples	*β* (k/min)	*T_p_* (k)	1000/*T_p_*	−*ln*(*β*/*T_p_*^2^)	*ln* *β*
CE/E51/8.0DEDDSQ	4	525.0	1.90	11.14	1.39
	6	541.5	1.85	10.79	1.79
	8	549.9	1.82	10.54	2.08
	10	558.0	1.79	10.35	2.30

**Table 3:** Activation energy and reaction levels for curing reaction of CE/E51/8.0DEDDSQ.

Kissinger Activation /kJ/mol	Ozawa Activation /kJ/mol	Average Activation Energy/kJ/mol	Crane Reaction Level *n*
58.77	67.75	63.26	1.07

**Table 4:** Data for pre-exponential factor *A*.

*β*	*A*	Average Value of *A*
4	2.19 × 10^5^	2.06 × 10^5^
6	1.97 × 10^5^	
8	2.05 × 10^5^	
10	2.04 × 10^5^	

**Table 5:** TGA data of CE/E51/DEDDSQ nanocomposites.

Sample	*T*_*d*,*max*_	Char Yield (%)
CE/E51	390.2	24.3
CE/E51/1.0DEDDSQ	394.1	26.1
CE/E51/1.5DEDDSQ	394.2	27.8
CE/E51/2.0DEDDSQ	394.7	28.3
CE/E51/5.0DEDDSQ	398.2	28.5
CE/E51/8.0DEDDSQ	402.6	29.1

## Data Availability

Data presented in this study are available on request from the corresponding author.

## References

[1] Liu Z., Zhang J., Tang L., Zhou Y., Lin Y., Wang R., Kong J., Tang Y., Gu J. (2019). Improved wave-transparent performances and enhanced mechanical properties for fluoride-containing PBO precursor modified cyanate ester resins and their PBO fibers/cyanate ester composites. Compos. Part B-Eng..

[2] Sreehari H., Sethulekshmi A.S., Saritha A. (2022). Bio epoxy coatings: An emergent green anticorrosive coating for the future. Macromol. Mater. Eng..

[3] Kumar D., Choudhary V. (2020). Curing kinetics and thermal properties of imide containing phthalonitrile resin using aromatic amines. Thermochim. Acta.

[4] Zhang J., Liu Z., Han M., Zhang J., Tang Y., Gu J. (2023). Block copolymer functionalized quartz fibers/cyanate ester wave-transparent laminated composites. J. Mater. Sci. Technol..

[5] Salunke A., Sasidharan S., Cherukattu Gopinathapanicker J., Kandasubramanian B., Anand A. (2021). Cyanate ester—Epoxy blends for structural and functional composites. Ind. Eng. Chem. Res..

[6] Tang L., Zhang J., Tang Y., Kong J., Liu T., Gu J. (2021). Polymer matrix wave-transparent composites: A review. J. Mater. Sci. Technol..

[7] Lin T., Zhang J., Junwei G. (2021). Random copolymer membrane coated PBO fibers with significantly improved interfacial adhesion for PBO fibers/cyanate ester composites. Chin. J. Aeronaut..

[8] Zhai L., Liu Z., Li C., Qu X., Zhang Q., Li G., Zhang X., Abdel-Magid B. (2019). Cyanate ester resin based composites with high toughness and thermal conductivity. RSC Adv..

[9] Jiang W., Zhang X., Chen D., Ma Y., Yang W. (2021). High performance low-k and wave-transparent cyanate ester resins modified with a novel bismaleimide hollow polymer microsphere. Compos. Part B-Eng..

[10] Fang L., Zhou J., He C., Tao Y., Wang C., Dai M., Wang H., Sun J., Fang Q. (2020). Understanding how intrinsic micro-pores affect the dielectric properties of polymers: An approach to synthesize ultra-low dielectric polymers with bulky tetrahedral units as cores. Polym. Chem..

[11] Li W., Huang W., Kang Y., Gong Y., Ying Y., Yu J., Zheng J., Qiao L., Che S. (2019). Fabrication and investigations of G-POSS/cyanate ester resin composites reinforced by silane-treated silica fibers. Compos. Sci. Technol..

[12] Yang H., He C., Russell T.P., Wang D. (2020). Epoxy-polyhedral oligomeric silsesquioxanes (POSS) nanocomposite vitrimers with high strength, toughness, and efficient relaxation. Giant.

[13] Sun D., Zhou Z., Chen G.-X., Li Q. (2014). Regulated dielectric loss of polymer composites from coating carbon nanotubes with a cross-linked silsesquioxane shell through free-radical polymerization. ACS Appl. Mater. Interfaces.

[14] Laird M., Van der Lee A., Dumitrescu D.G., Carcel C., Ouali A., Bartlett J.R., Unno M., Wong Chi Man M. (2020). Styryl-functionalized cage silsesquioxanes as nanoblocks for 3-D assembly. Organometallics.

[15] Xu Z., Zhao Y., Wang X., Lin T. (2013). A thermally healable polyhedral oligomeric silsesquioxane (POSS) nanocomposite based on Diels–Alder chemistry. Chem. Commun..

[16] Miao L., Zhan L., Liao S., Li Y., He T., Yin S., Wu L., Qiu H. (2024). The Recent Advances of Polymer–POSS Nanocomposites With Low Dielectric Constant. Macromol. Rapid Comm..

[17] Zhao X., Liu H. (2024). Hybrid porous polymers based on double-decker and cage-ype silsesquioxanes for the high-efficiency removal of neonicotinoid insecticides and dyes. J. Polym. Sci..

[18] Chen C.-Y., Mohamed M.G., Chen W.C., Kuo S.-W. (2023). Construction of Ultrastable porous carbons materials derived from organic/inorganic double decker silsesquioxane (DDSQ) hybrid as a high-performance electrode for supercapacitor. Mater. Today Chem..

[19] Hirosawa Y., Imoto H., Naka K. (2024). Polymers with double-decker and side-opened cage silsesquioxanes: Effects of cage structures on materials properties. J. Polym. Sci..

[20] John Ł., Ejfler J. (2023). A brief review on selected applications of hybrid materials based on functionalized cage-like silsesquioxanes. Polymers.

[21] Qiu J., Xu S., Liu N., Wei K., Li L., Zheng S. (2018). Organic–inorganic polyimide nanocomposites containing a tetrafunctional polyhedral oligomeric silsesquioxane amine: Synthesis, morphology and thermomechanical properties. Polym. Int..

[22] Liu N., Li L., Wang L., Zheng S. (2017). Organic-inorganic polybenzoxazine copolymers with double decker silsesquioxanes in the main chains: Synthesis and thermally activated ring-opening polymerization behavior. Polymer.

[23] Zhao H., Zhao S.-Q., Li Q., Khan M.R., Liu Y., Lu P., Huang C.-X., Huang L.-J., Jiang T. (2020). Fabrication and properties of waterborne thermoplastic polyurethane nanocomposite enhanced by the POSS with low dielectric constants. Polymer.

[24] Xu R., Liu K., Zhang Y., Feng X., Yu D., Ishida H., Froimowicz P. (2017). Recent Progress in Polybenzoxazine/POSS Hybrid Materials. Advanced and Emerging Polybenzoxazine Science and Technology.

[25] Kurinchyselvan S., Hariharan A., Prabunathan P., Gomathipriya P., Alagar M. (2019). Fluorinated polyimide nanocomposites for low K dielectric applications. J. Polym. Res..

[26] Luo K., Song G., Wang Y., Yu J., Zhu J., Hu Z. (2019). Low-k and recyclable high-performance POSS/polyamide composites based on Diels–Alder reaction. ACS Appl. Polym. Mater..

[27] Chen W.-C., Tsao Y.-H., Wang C.-F., Huang C.-F., Dai L., Chen T., Kuo S.-W. (2020). Main chain–type block copolymers through atom transfer radical polymerization from double-decker–shaped polyhedral oligomeric silsesquioxane hybrids. Polymers.

[28] Mohamed M.G., Kuo S.W. (2019). Functional silica and carbon nanocomposites based on polybenzoxazines. Macromol. Chem. Phys..

[29] Chen W.-C., Kuo S.-W. (2018). Ortho-imide and allyl groups effect on highly thermally stable polybenzoxazine/double-decker-shaped polyhedral silsesquioxane hybrids. Macromolecules.

[30] Joseph A.M., Nagendra B., Surendran K., Gowd E.B. (2019). Sustainable in situ approach to covalently functionalize graphene oxide with POSS molecules possessing extremely low dielectric behavior. Langmuir.

[31] Ma Y., He Z., Liao Z., Xie J., Yue H., Gao X. (2021). Facile strategy for low dielectric constant polyimide/silsesquioxane composite films: Structural design inspired from nature. J. Mater. Ssi..

[32] Guo M., David É., Fréchette M., Demarquette N.R. (2017). Polyethylene/polyhedral oligomeric silsesquioxanes composites: Dielectric, thermal and rheological properties. Polymer.

[33] Zhang X., Liu M., Chen Y., He J., Wang X., Xie J., Li Z., Chen Z., Fu Y., Xiong C. (2022). Epoxy resin/hollow glass microspheres composite materials with low dielectric constant and excellent mechanical performance. J. Appl. Polym. Sci..

[34] Li X., Liu X., Feng J., He R., Liu H., Hu X., Liu X. (2023). A research on benzoxazine/cyanate ester/epoxy POSS nanocomposite with low dielectric constant and improved toughness. Polym. Bull..

[35] Liu X., Zhou J., Zhou Y., Wu M., Zhu Y., Zhao J., Liu S., Xiao H. (2022). Chemically crosslinked polyimide-POSS hybrid: A dielectric material with improved dimensional stability and dielectric properties. Eur. Polym. J..

[36] Zhang Z., Zhou Y., Cai L., Xuan L., Wu X., Ma X. (2022). Synthesis of eugenol-functionalized polyhedral oligomer silsesquioxane for low-k bismaleimide resin combined with excellent mechanical and thermal properties as well as its composite reinforced by silicon fiber. Chem. Eng. J..

[37] Periyasamy T., Asrafali S.P., Muthusamy S. (2015). New benzoxazines containing polyhedral oligomeric silsesquioxane from eugenol, guaiacol and vanillin. New J. Chem..

[38] Li X., Liu X., Liu H., He R., Wang F., Meng S., Li Z. (2023). The low-k epoxy/cyanate nanocomposite modified with epoxy-based POSS: The effect of microstructure on dielectric properties. React. Funct. Polym..

[39] Wang C., Feng Y., Zhang C., Zhang T., Chi Q., Chen Q., Lei Q. (2022). High heat-resistant (250 °C) epoxy resin composites with excellent dielectric properties. J. Appl. Polym. Sci..

[40] Zhang C., Liu N., Li L., Wang L., Zheng S. (2017). Polybenzoxazine nanocomposites containing 3, 13-Diglycidyl double-decker silsesquioxane. Polym. Compos..

[41] Sun X., Wang J., Fu Q., Zhang Q., Xu R. (2022). Synthesis of a novel bifunctional epoxy double-decker silsesquioxane: Improvement of the thermal stability and dielectric properties of polybenzoxazine. Polymers.

[42] Zhang P., Zhang K., Chen X., Dou S., Zhao J., Li Y. (2021). Mechanical, dielectric and thermal properties of polyimide films with sandwich structure. Compos. Struct..

[43] (2008). Plastics—Determination of Water Absorption.

